# Relationship between Nutrition and Development of the Jaws in Children: A Pilot Study

**DOI:** 10.3390/children11020201

**Published:** 2024-02-05

**Authors:** Paula Boo Gordillo, Laura Marqués Martínez, Carla Borrell García, Esther García Miralles

**Affiliations:** 1 Doctoral School, Faculty of Medicine and Health Sciences, Catholic University of Valencia, San Vicente Martir, 46001 Valencia, Spain; paula.boo@ucv.es; 2 Faculty of Medicine and Health Sciences, Catholic University of Valencia, San Vicente Martir, 46001 Valencia, Spain; carla.borrell@ucv.es (C.B.G.); esther.garcia@ucv.es (E.G.M.)

**Keywords:** feeding, early childhood, temporary dentition, pacifier, occlusion

## Abstract

Craniofacial growth and development have been shown to be influenced by various environmental factors that impact child development. This study aims to analyze the different patterns of feeding during early childhood, starting from birth, and assess the variability of nutrition during the first stage of childhood, along with the habits developed, to study their impact on jaw development. The study was conducted on a sample of twenty-five patients aged 3 to 5, following approval from the ethics committee of the Catholic University of Valencia. Informed consent was obtained from the fathers, mothers, and/or legal guardians, who were administered surveys on habits and diet. Cephalometric measurements within the parameters of ideal occlusion were subsequently taken. While previous studies examined this subject, the findings are challenging to evaluate. However, this study identified significant associations (*p* = 0.001) between clinical measurements and children’s eating habits. The growth and development of the craniofacial cavity are influenced by multiple factors, including a child’s diet and habits. Nonetheless, further research is required to determine whether diet can be considered a determining factor in proper jaw growth.

## 1. Introduction

The development of dentition is intricately linked and coordinated with jaw growth. It initiates during intrauterine life with the calcification of primary teeth, followed by their eruption, subsequent exfoliation, and eventually the eruption of permanent teeth [1]. Jaw growth exhibits individual variations largely influenced by genetic factors [2]. However, it is widely accepted that the variability in dental occlusion results from a combination of multiple factors involving both genetic and environmental influences [3,4,5,6].

Occlusion is defined as the intercuspation of the upper and lower teeth when the jaws are in occlusion, which means when they are static [7,8,9,10,11,12]. Primary teeth significantly affect the growth and development of a child’s dentition [13,14,15,16,17,18,19,20]. Proper occlusion of the teeth plays an important role in esthetics, as well as in chewing, swallowing, speaking, and breathing and promotes normal function and growth. Primary teeth ensure the eruption of permanent successors in their position and time. Therefore, the development of a normal occlusion is essential to overall good health [21,22,23,24,25,26,27,28,29].

The normal occlusion of deciduous teeth is characterized by the following clinical features [30,31,32,33,34,35,36,37]:

- Presence of spaces between the four upper and lower incisors.
- Primate spaces in mesial of maxillary canines and distal of mandibular canines.
- Straight terminal step, where the distal face of the lower second primary molar is at the same level as the distal face of the upper second primary molar.
- Overbite, which is the vertical overlap or the amount of space in millimeters that the upper central incisor covers the lower central incisor. A normal overbite is generally considered to be in the range of 2–3 mm [30,31,32,33,34,35,36].
- Overjet, which is the space in millimeters between the buccal face of the lower central incisor and the palatal face of the upper central incisor. A normal overjet is considered 2–3 mm.

The duration of breastfeeding has been a subject of great scientific interest [10]. According to the World Health Organization (WHO), breastfeeding must be exclusive until 6 months of age due to its innumerable benefits for both the child and the mother [11,12,13,14,15,16,17].

Breastfeeding is recognized as a crucial and fundamental factor for the proper growth and development of the body, including the stomatognathic and orofacial musculature. It is considered the optimal orthopedic treatment that promotes the achievement of a harmoniously developed adult face. Breastfeeding plays a vital role in the proper development of the entire craniofacial complex, particularly during the critical stages of a child’s life [18,21].

The upward and outward forces generated by the tongue during sucking have a significant impact on the growth of the infant’s premaxilla region, while the movements of the mandible stimulate mandibular growth [19]. Extensive research in the literature demonstrated that prolonged breastfeeding plays a crucial role in preventing the development of malocclusion, enhancing the sagittal growth of the mandible, and establishing proper occlusal relationships by stimulating the facial muscles during lactation.

Different authors hold contrasting views on the significance of diet versus heredity in the development of facial and occlusal characteristics. Some argue that diet plays the most crucial role, relegating heredity to a secondary role, while others consider genetic influence to be the primary determining factor [20,22].

An important aspect when it comes to jaw development is the presence of low birth weight, which, according to the World Health Organization (WHO), is defined as a birth weight of less than 2500 g [23].

Overweight and obesity are defined as an abnormal or excessive accumulation of fat that can be harmful to health. Body mass index (BMI) is a simple indicator of the relationship between weight and height that is frequently used to identify overweight and obesity in adults. It is calculated by dividing a person’s weight in kilos by the square of their height in meters (kg/m^2^) [24].

The prevalence of overweight and obesity in children and adolescents (aged 5 to 19) has increased dramatically, from 4% in 1975 to more than 18% in 2016. This increase has been similar in both sexes: 18% of girls and 19% of boys were overweight in 2016 [24].

### Habits during Childhood

As previously mentioned, early sucking activity has an impact on the growth of the craniofacial complex. Research indicates that non-nutritive sucking habits, such as pacifier use or thumb-sucking, can contribute to certain forms of malocclusion, particularly open bite and posterior crossbite [25]. Pacifier use is prevalent among infants and children globally. Pacifiers are frequently utilized to soothe crying babies, promote well-being for both infants and parents, and discourage thumb or finger sucking [25,26].

In certain developed countries, pacifier use has become deeply ingrained in the culture, leading to a high prevalence of up to 42.5% among young children at the age of 12 months [27]. Non-randomized studies indicated that the use of conventional pacifiers can negatively affect the development of orofacial structures, potentially leading to infections, a shortened duration of lactation, and the development of dental malocclusions [26,28].

It was reported that the use of pacifiers may have a protective effect against sudden infant death syndrome (SIDS), although the level of evidence supporting this claim is currently very low [26].

The effects of pacifier use are dependent on its duration and frequency [29]. Certain studies indicate that during non-nutritive habits, the forces generated by the buccinator muscles are directed toward the posterior area. It was observed that these forces can restrict the development of the mandible and promote disto-occlusion [22].

In the case of bottle-feeding, its impact is similar to what was described above. Studies demonstrated that bottle-fed children are more likely to suffer posterior crossbite compared to breastfed children [25,30,31].

The different involvement of the muscles and the different impact on the palate during the sucking of the bottle is presumably responsible for the misalignment of the teeth and the abnormal transverse growth of the palate, conditions that lead to a posterior crossbite [25,27,30].

By studying occlusion in temporary dentition and the environmental factors that influence its correct development, it is possible to understand the importance and complexity of the correct development of the occlusion.

Due to the scarcity of studies in our geographical context, the present study seeks to be able to establish, in the future, a guide for the introduction of food during childhood that favors the correct function of the musculature and adequate development of the jaws.

In this study, our primary focus was on investigating the influence of diet on jaw development. By analyzing the environmental factors that may contribute to changes in occlusion in primary dentition, the literature suggests that a lower prevalence of occlusal and proximal tooth wear is associated with a higher incidence of crowding. Therefore, the objective of this study is to examine the relationship between feeding patterns, the presence of abnormal habits, and the proper development of the jaws in children aged 3 to 5 years.

## 2. Materials and Methods

This study was approved by the ethics committee of the Catholic University of Valencia on 1 February 2022, with project code UCV/2021-2022/094.

Inclusion criteria:

Patients aged 3 under 6 since they have full temporary dentition.

Patients in full temporary dentition.

Healthy children with no diagnosis of syndromes or systemic pathologies associated with oral or facial alterations.

Patients whose father/mother or legal guardian provided signed consent for their participation in the study.

Patients whose father/mother or legal guardian completed the survey on eating and habits completely and correctly.

Exclusion criteria:

Children with any premature tooth loss.

Children wearing any kind of orthodontic appliance or space maintainer.

Uncooperative children who are not able to be measured and recorded.

Once it was confirmed that each patient met all the inclusion criteria and did not meet any exclusion criteria, the legal guardian of the patient was provided with the study information sheet and informed consent for compliance. Subsequently, they were provided with an anonymous survey to be completed accurately in order to gather the necessary information required for the study.

The main questions included in the survey were

- Breastfeeding: Frequency and duration of breastfeeding, or in the case of artificial milk feeding, the duration of the period in which this type of milk was used.
- Bottle usage: Age at which the child started using a bottle and the frequency of its use.
- Pacifier usage: Age at which the child started using a pacifier and the frequency of its use (with possible answers being “during all day”, “only when the child is at home”, or “only for sleep time”).
- Introduction of supplementary feeding: Age at which supplementary feeding was introduced and the method of introduction.
- Weekly food intake: Parents were asked to classify foods into liquids, semi-solids/puree, and solids, based on the child’s five daily meals (breakfast, lunch, snack, dinner, pre-bed intake). This information was to be recorded in a three-column table.

After collecting survey data, measurements were taken on the patients participating in the study. These measurements were conducted by an expert in the field using a vernier caliper to obtain the necessary data in millimeters. The recorded measurements included the following:

- Interincisal diastemas: Obtained by summing the spaces between the temporary central incisors and temporary lateral incisors in millimeters. These measurements were taken in both the upper and lower arches.
- Primate spaces: This measurement required assessing the distance from the distal aspect of the temporary lateral incisor to the mesial aspect of the temporary canine. Thus, for both arches, four sets of data were obtained corresponding to the upper right quadrant, upper left quadrant, lower right quadrant, and lower left quadrant.
- Overjet: These data were obtained by measuring the distance in millimeters between the facial surface of the temporary lower incisors and the palatal surface of the temporary upper incisors.
- Overbite: Obtained by measuring the space in millimeters that the temporary upper incisor covers over the temporary lower incisor.

After taking these measurements, the expert clinically classified the patient’s occlusion through clinical examination:

- Straight terminal plane: When the distal surfaces of the temporary second molars coincide in the same vertical plane.
- Mesial step: When the distal surface of the mandibular second molar is anterior to that of the maxillary second molar.
- Distal step: When the distal surface of the mandibular second molar is posterior to that of the maxillary second molar.

All measured values were recorded on an anonymous patient identification data collection sheet.

The Mann–Whitney test for two independent samples was used to perform the statistical analysis of the sample in order to contrast whether the distribution of a variable, at least ordinal, is the same in two independent samples, as defined by the affirmative and negative responses to a questionnaire question (e.g., breastfeeding).

Extending Kruskal–Wallis to more than two samples was also used.

Spearman’s nonlinear correlation coefficient was used as well, with a significance level of 5% (α = 0.05).

A Mann–Whitney test reaches a power of 50% to detect as significant a large effect size (d = 0.8) in the comparison of the distribution of a parameter in 2 independent groups, with a confidence level of 95%.

The sample size for this pilot study was determined to be 25 subjects, which will be used to analyze and obtain the Intra-examiner Kappa value. These values are supported by the Babbie authors. E. and Garcia-Garcia JA et al. [32,33] recommend including a similar number of subjects for pilot studies. These subjects must possess the attributes that are desired to be measured in the subject population.

The conduct of this pilot study arises from the need to validate the survey that parents had to fill out before measuring oral parameters with the objective of analyzing whether the questions were understandable and easily answered by parents and their relationship with oral measurements.

Before carrying out the study, the anonymous survey was prepared by the main researcher and reviewed by 3 dentistry professors and 2 nutrition degree professors who were experts in the subject and who provided their modifications and suggestions.

## 3. Results

The research sample consisted of 25 children aged between 3 and 5, including 14 boys (56%) and 11 girls (44%).

Among the cases of breastfeeding (84%), it was exclusive until 6 months in 14.3%; otherwise, it lasted beyond 6 months in 94.4%.

**Figure 1:**
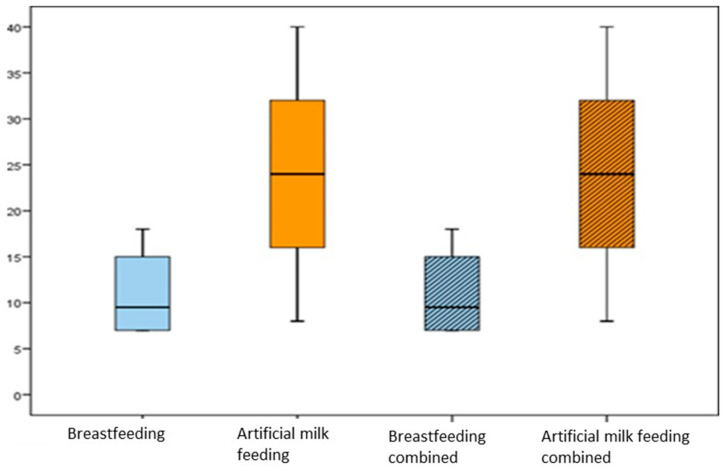
Age of the child at the different moments of completion of a feeding stage.

The median age at the cessation of breastfeeding was 24 months (Figure 1), with an interquartile range (IQR) of 12 to 30. In 61.1% of the cases, breastfeeding was offered on demand by the mothers.

Regarding artificial milk feeding (48%), it was exclusive until 6 months for 25 of the children, while for the rest, it was continued beyond 6 months. The median age at the end of artificial milk feeding was 18 months (Figure 1), with an IQR of 12–24.

In 24% of the cases, a combination of both breastfeeding and artificial milk feeding was observed. For these children, the median age at the cessation of breastfeeding was 9.5 months (Figure 1), with an IQR of 7 to 18, while the median age at the end of artificial milk feeding was 21 months (Figure 1), with an IQR of 8 to 24.

In order to establish whether the development of the jaws was correct, cephalometric parameters representing the ideal occlusion were established based on the literature reviewed.

**Figure 2:**
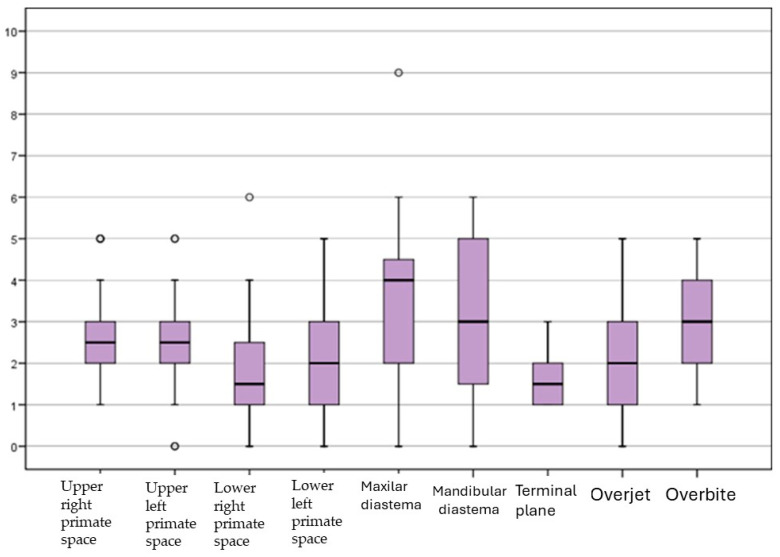
Means obtained from the measurements of the cephalometric parameters.

The mean results (Figure 2) obtained among the 25 participants were as follows:

- Primate space: Medians were 2.7 mm and 2.5 mm for the upper sides and 1.7 and 1.8 mm for the lower ones.
- Interincisive diastema: 3.3 mm maxilar and 3.0 mm mandibular.
- Terminal plane: Median 1.7 (flush terminal plane predominated)
- Overjet: 2.0 mm.
- Overbite: 3.0 mm.

In Table 1, we can observe the results for participants who received breastfeeding as a method of feeding.

A moderate and significant correlation (r = −0.55, *p* = 0.019) was found between the measure of the terminal plane and the age of completion of breastfeeding. It was observed that a later age of completion of breastfeeding was associated with lower values of the terminal plane, indicating a higher prevalence of a flush terminal plane.

Among the patients who were fed with artificial milk, a significant difference was observed in the measure of the right lower primate space (*p* = 0.016) (Table 2). This dimension was found to be reduced in cases where children received exclusive artificial milk feeding.

In the case of combined feeding (breastfeeding + artificial milk), a strong correlation was observed between the age of weaning and the overbite measurement (r = −0.93; *p* = 0.008) (Table 3). It was noted that the earlier the baby was weaned from breastfeeding and introduced to artificial milk, the greater the overbite measurements.

The rate of bottle use among the participants was 72%. The median age at which bottle use ended was 24 months (IQR: 12–36).

**Figure 3:**
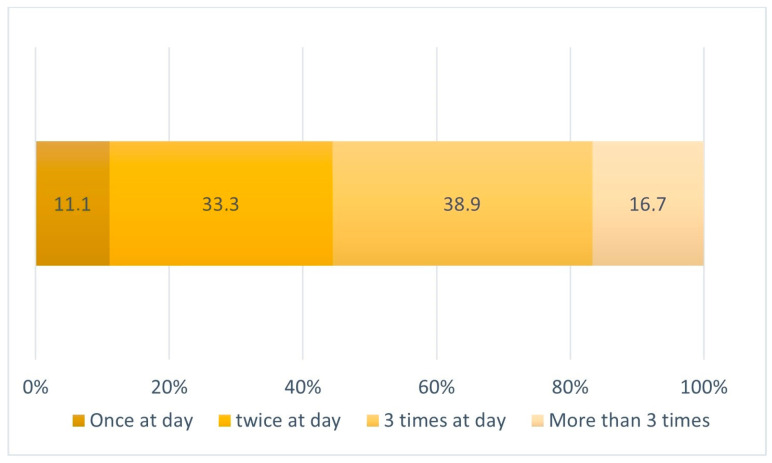
Frequency of daily bottle feedings.

Figure 3 provides information on the times per day when the bottle was taken. It highlights that the bottle was commonly used during breakfast (88.9%), dinner (55.6%), recena (evening snack) (50%), and night shots (44.4%). When the total number of daily bottle shots is added, the following distribution can be established (Figure 3).

According to Table 4, no significant correlations were found between cephalometric values and bottle use. The strongest correlation was observed between overbite and the number of bottles per day, with a correlation coefficient of −0.46 (*p* = 0.067). This suggests that parameter measurement tends to be larger in children who consumed fewer bottles per day, although the relationship did not reach statistical significance.

**Figure 4:**
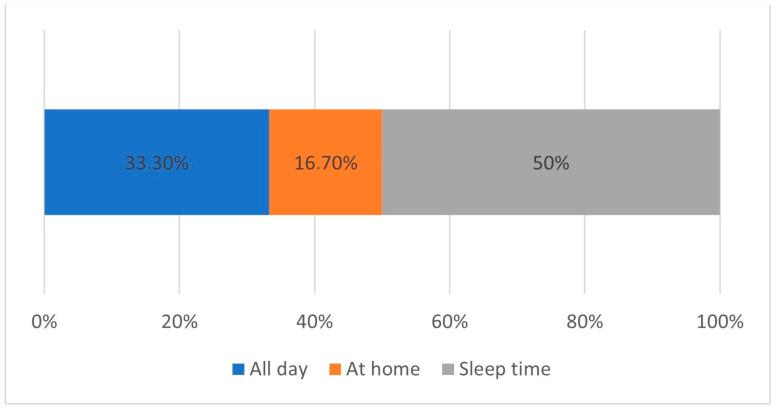
Frequency of pacifier use.

In terms of the frequency of pacifier use (Figure 4), 50% of the patients reported using it only during the night.

Table 5 indicates a significant correlation (*p* = 0.035) between the measurement of the upper primate space and pacifier use. Additionally, there is a weak tendency (*p* = 0.098) observed in the primate space on the left side.

These findings suggest that pacifier use may contribute to a smaller physiological space.

**Figure 5:**
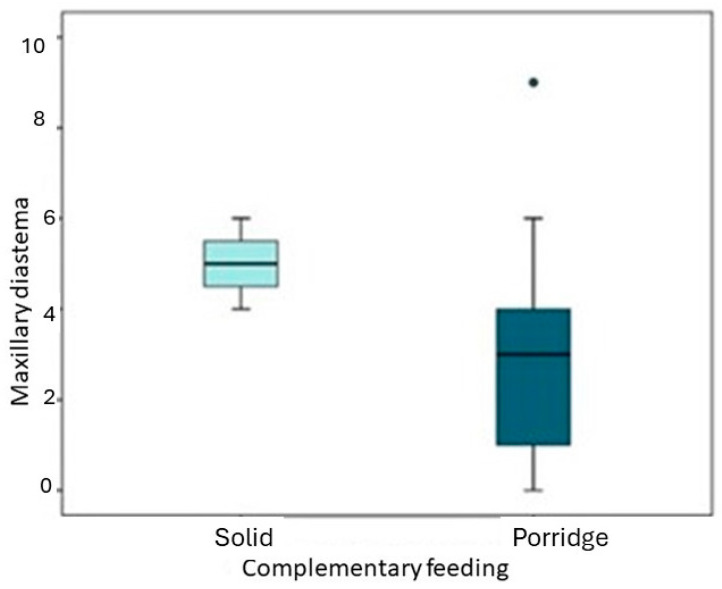
Association between the type of complementary feeding and maxillary diastema.

Significant differences were observed (Table 6) when comparing the age of initiation of complementary feeding and the type of feeding (porridge or solids). Specifically, the distribution of maxillary diastema (gap between the upper incisors) differed significantly between the two groups (Figure 5). The children who began complementary feeding with solid foods had a larger diastema compared to those who started with porridge.

Furthermore, an enlargement of the right lower primate space (gap between certain teeth) was detected when complementary feeding was initiated at an earlier age. Although the association is weaker, the data suggest that an earlier introduction of complementary feeding is associated with values indicating a flush terminal plane, meaning a more even alignment of the back teeth.

These findings suggest that the type and timing of complementary feeding, particularly the introduction of solid foods, may have an impact on dental development, specifically in relation to maxillary diastema and the alignment of the back teeth.

The study also observed that an early introduction of complementary feeding was associated with an enlargement of the right lower primate space and that there is a weaker but still noticeable association between the early introduction of complementary feeding and values indicating a flush terminal plane.

The study revealed that half of the sample consumed solids more than 28 times per week (median = 28), and 25% of the participants indicated consuming solids more than 35 times per week.

Several significant relationships were detected (Table 7). The overjet showed a moderate to strong inverse association with fluid intake (*p* = 0.001), indicating that higher weekly fluid consumption was associated with lower overjet values. A similar interpretation was observed for the number of times semi-solids/purees were consumed. On the other hand, a higher frequency of solid food intake seemed to be associated with a narrower diastema.

These findings suggest that the amount and type of food consumed weekly may have an influence on the development of the jaws, as indicated by the cephalometric parameters analyzed in this study.

**Figure 6:**
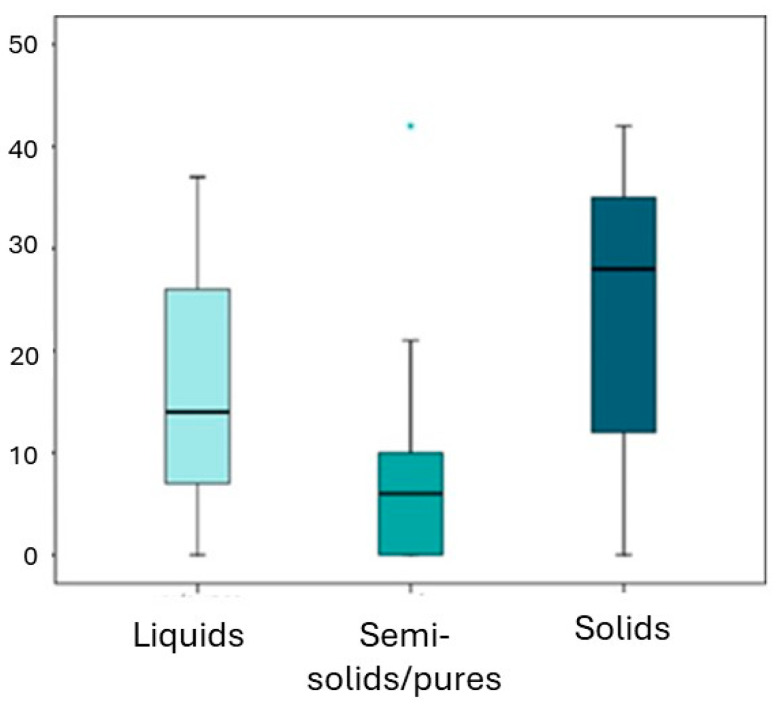
Distribution of the weekly amount by type of food.

Finally, the main objective of this study was to establish a relationship between the amount of liquids, semi-solids and/or purees and solids consumed by participants weekly (Figure 6) and to be able to relate if the variation of these intakes affects the correct development of the jaws based on the cephalometric parameters explained above.

Half of the sample was found to consume solids more than 28 times a week (median = 28), and 25% of the sample indicated that they consumed solids more than 35 times.

Some significant relationships were detected (Table 7). The overjet has an inverse association of medium–strong magnitude with fluid intake (*p* = 0.001). The greater the weekly fluid consumption, the lower the value of the overjet. For the number of semi-solids, the interpretation was similar. On the other hand, the lower diastema seems to increase if the number of times solids are taken is very high.

## 4. Discussion

The duration of breastfeeding in children has garnered significant scientific interest [11]. Breastfeeding serves a purpose beyond providing mere nutrition; it plays a crucial and fundamental role in promoting the proper growth and development of the body, including the stomatognathic and orofacial muscles. Several authors endorse the notion that the tongue’s upward and outward forces during suction influence the growth of the child’s premaxilla region, while mandibular movements stimulate mandibular growth [22].

Certain authors advocate for the significance of extending the duration of breastfeeding to prevent the development of malocclusion, enhance sagittal jaw growth, and establish a proper occlusal relationship by stimulating the muscles [38]. This study observed that children who continue breastfeeding beyond six months of age exhibit a higher prevalence of a straight terminal step. The development of a straight terminal plane leads to a Class I Angle in the permanent dentition, attributable to a combination of jaw advancement and the mesial migration of the mandibular arch [39]. Additionally, a study conducted in Beijing by Chen et al. revealed that children who either did not receive breastfeeding or breastfed for less than six months were more susceptible to subsequent crossbite, which is directly linked to the position of the terminal plane [40]. The literature states that bottle-feeding for more than 18 months is associated with a higher prevalence of a tendency toward a distal terminal plane [38,41].

Ravn et al. demonstrated that a distal staggered molar relationship has the potential to progress into a Class II molar relationship according to Angle’s classification [42]. Another study focused on children who were bottle-fed for over 18 months revealed that they had a 1.45 times higher risk of lacking a mesial step and a 1.43 times higher risk of developing a Class II canine relationship in comparison to children who were bottle-fed for 6–18 months [42].

It is important to note that our study did not find any significant correlations between the terminal plane and the use of a bottle. However, the most noteworthy relationship was observed between an increase in overbite and the frequency of daily bottle usage. Children who consumed multiple bottles per day tended to have a larger measurement for this parameter.

In this study, when referring to non-nutritive habits, we specifically mean the use of pacifiers. A separate study demonstrated that using a pacifier for more than 36 months was associated with the development of anterior open bite malocclusion (AOB) [42].

Warren and Bishara et al. conducted a study revealing that children who used a pacifier for over 2 years had a higher likelihood of developing anterior open bite (AOB), characterized by a negative overjet and overbite. In contrast, children who used a pacifier for a shorter duration of 2 years or less exhibited overbite and overjet values within the average range (approximately 2–3 mm, respectively) [43].

Similarly, it was observed that a prolonged duration of pacifier use leads to a statistically significant decrease in palatal depths, which promotes the compression of the maxillary arch [44].

However, another recent study demonstrated that the duration of pacifier use could influence the association between a longer duration of breastfeeding and a lower prevalence of AOB [45].

In another study conducted by Chen et al., when examining the sucking habit of children with pacifiers, a significant association was found between the absence of a physiological diastema space and a decreased lower arch [40].

This study demonstrates that pacifier use is associated with the reduced development of the primate space and interincisive diastema. Consequently, a diminished physiological space increases the susceptibility to crowding and malocclusion in permanent dentition [40].

In this study, the average age for the introduction of complementary feeding was found to be 9 months, with porridge being the initial food offered. While the literature does not demonstrate a direct relationship between the introduction of solid foods and cephalometric parameters, an association was identified between starting solid foods and the presence of larger physiological spaces. This suggests that initiating feeding with solid foods may lead to a lower tendency for crowding. Primate spaces and interincisive diastemas are considered essential and important as they facilitate the proper alignment of permanent teeth by occupying the necessary space for a harmonious occlusion [46].

The role of early feeding in occlusion seems unclear based on the published results, and further assessment is needed to gain a better understanding.

Our primary objective was to investigate whether there is a relationship between childhood feeding patterns and the proper development of the jaws. It was observed that children whose diet mainly consists of liquids and/or semi-solid foods experience a decrease in physiological spaces concurrently with a decrease in overjet values. Conversely, if a solid diet is predominant, the measurements of physiological diastemas, particularly in the lower arch, tend to increase. Mastication plays a significant role in tooth wear and stimulates bone development through chewing, contributing to the correct development of the jaws. Ideal occlusion factors such as interincisive diastemas and primate spaces are associated with proper jaw development [7,8].

Relating parental knowledge about nutrition and occlusion, findings of the study of Duraisamy revealed that feeding patterns were not associated with the prevalence of malocclusion. Mothers in the study had basic knowledge of feeding practices and their association with caries and malocclusion. However, their theoretical knowledge was not fully reflected in the way they cared for their children’s teeth. Longitudinal studies assessing the relationship between malocclusion and different feeding methods considering the phenotypes of parents and their children would provide useful data on the perception of the etiology of analyzed malocclusion [47].

Currently, the literature has not yet described whether specific dietary patterns favor the parameters established for ideal occlusion. Therefore, further studies are necessary to determine which types of diets promote the proper development of the jaws and help prevent future functional problems.

As this is a pilot study, the sample size was reduced. This sample size is one of the limitations of our research. We also know that there are certain factors that must also be considered, such as genetic or environmental factors, low birth weight, and premature birth. Furthermore, it would have been important to deepen and relate the parents’ knowledge about nutrition and occlusion. As for strengths, it is difficult to measure such young patients, and this is a novel study since we found few articles that talk about this topic, and we analyze several factors that can alter occlusion, such as bottle-feeding and pacifiers and their duration and frequency.

## 5. Conclusions

In conclusion, significant associations were identified between clinical measurements and the feeding habits of children.

Breastfed children tend to exhibit more favorable ideal occlusion values compared to those who were fed artificial milk. Furthermore, when complementary feeding begins with solid foods, there is a higher likelihood of finding superior diastemas, and an earlier introduction to solid foods is associated with better ideal occlusion values.

The use of pacifiers was observed to reduce upper primate spaces, while a higher frequency of intake of liquids or semi-solid foods is linked to increased overjet values. Conversely, the introduction of solid foods tends to enlarge mandibular diastemas.

Further research is necessary to establish guidelines for the introduction of complementary foods during childhood that promote the proper development of the jaws.

## Figures and Tables

**Table 1:** Association between clinical parameters and breastfeeding variables: Mann–Whitney test results and Spearman’s r correlation coefficient.

		On-Demand	Age of Completion of Breastfeeding
upper right primate	0.452	1.00	r = 0.27; *p* = 0.271
upper left primate	0.452	0.659	r = 0.29; *p* = 0.240
lower right primate space	0.543	0.425	r = 0.41; *p* = 0.090
lower left primate space	0.543	0.930	r = 0.14; *p* = 0.583
maxilar diastema	0.858	0.930	r = 0.04; *p* = 0.874
mandibular diastema	0.592	0.375	r = 0.15; *p* = 0.557
terminal plane	0.971	0.375	r = −0.55; *p* = 0.019 *
overjet	0.543	0.536	r = −0.26; *p* = 0.293
overbite	0.477	0.791	r = −0.40; *p* = 0.097

* *p* < 0.05.

**Table 2:** Association between clinical parameters and variables of lactation by artificial milk: Mann–Whitney test results and Spearman’s r correlation coefficient.

	Lactation by Artificial Milk	Age of Completion of Feeding
upper right primate	0.728	r = 0.24; *p* = 0.572
upper left primate	0.295	r = −0.19; *p* = 0.619
lower right primate space	0.016 *	r = −0.28; *p* = 0.460
lower left primate space	0.205	r = 0.00; *p* = 1.00
maxilar diastema	0.168	r = −0.40; *p* = 0.285
mandibular diastema	0.376	r = 0.28; *p* = 0.460
terminal plane	0.538	r = 0.24; *p* = 0.540
overjet	0.538	r = −0.04; *p* = 0.918
overbite	0.733	r = −0.04; *p* = 0.918

* *p* < 0.05.

**Table 3:** Association between clinical parameters and variables of combined breastfeeding: Mann–Whitney test results and Spearman’s r correlation coefficient.

	Combined Breastfeeding	Age of Completion of Breastfeeding	Age of Completion of Artificial Milk
upper right primate	0.780	r = 0.31; *p* = 0.545	r = −0.40; *p* = 0.428
upper left primate	0.333	r = 0.34; *p* = 0.506	r = −0.60; *p* = 0.208
lower right primate space	0.080	r = 0.20; *p* = 0.699	r = −0.75; *p* = 0.085
lower left primate space	0.733	r = 0.41; *p* = 0.417	r = −0.38; *p* = 0.454
maxilar diastema	0.555	r = 0.03; *p* = 0.955	r = −0.27; *p* = 0.607
mandibular diastema	0.733	r = −0.49; *p* = 0.321	r = −0.49; *p* = 0.321
terminal plane	0.877	r = −0.17; *p* = 0.744	r = 0.41; *p* = 0.423
overjet	0.555	r = −0.40; *p* = 0.428	r = −0.45; *p* = 0.373
overbite	0.090	r = −0.93; *p* = 0.008 **	r = −0.09; *p* = 0.866

** *p* < 0.01.

**Table 4:** Association between clinical parameters and bottle-feeding variables: Mann–Whitney test results and Spearman’s r correlation coefficient.

	Bottle-Feeding	Age of Completion of Bottle-Feeding	Number of Bottle-Feeding
upper right primate	0.357	r = 0.31; *p* = 0.545	r = 0.24; *p* = 0.347
upper left primate	0.270	r = 0.02; *p* = 0.928	r = −0.34; *p* = 0.166
lower right primate space	0.074	r = 0.21; *p* = 0.398	r = −0.04; *p* = 0.881
lower left primate space	0.657	r = 0.31; *p* = 0.204	r = −0.26; *p* = 0.294
maxilar diastema	0.326	r = 0.14; *p* = 0.577	r = 0.22; *p* = 0.364
mandibular diastema	0.615	r = 0.43; *p* = 0.075	r = 0.01; *p* = 0.970
terminal plane	0.976	r = 0.01; *p* = 0.962	r = −0.25; *p* = 0.312
overjet	0.362	r = −0.08; *p* = 0.756	r = −0.46; *p* = 0.067
overbite	0.099	r = −0.15; *p* = 0.556	r = −0.09; *p* = 0.866

**Table 5:** Association between clinical parameters and pacifier variables: Mann–Whitney test results and Spearman’s r correlation coefficient.

	Pacifier	Age of Completion of Pacifier	Use Time
upper right primate	0.035 *	r = 0.05; *p* = 0.890	0.683
upper left primate	0.098	r = 0.04; *p* = 0.909	0.683
lower right primate space	0.168	r = −0.40; *p* = 0.202	0.461
lower left primate space	0.137	r = −0.31; *p* = 0.321	0.808
maxilar diastema	0.225	r = 0.28; *p* = 0.376	0.683
mandibular diastema	0.247	r = 0.39; *p* = 0.206	0.154
terminal plane	0.347	r = 0.13; *p* = 0.689	0.461
overjet	0.247	r = −0.17; *p* = 0.601	0.214
overbite	0.135	r = −0.17; *p* = 0.626	0.279

* *p* < 0.05.

**Table 6:** Association between clinical parameters and complementary feeding variables: Mann–Whitney test results and Spearman’s r correlation coefficient.

	Type of Feeding	Age of Initiation
upper right primate	0.262	r = −0.14; *p* = 0.515
upper left primate	0.409	r = −0.02; *p* = 0.910
lower right primate space	0.268	r = −0.47; *p* = 0.019 *
lower left primate space	0.748	r = −0.17; *p* = 0.431
maxilar diastema	0.031 *	r = −0.08; *p* = 0.709
mandibular diastema	0.331	r = 0.07; *p* = 0.742
terminal plane	0.497	r = 0.41; *p* = 0.041 *
overjet	0.971	r = 0.31; *p* = 0.136
overbite	1.00	r = 0.16; *p* = 0.470

* *p* < 0.05.

**Table 7:** Association between clinical parameters and amount of weekly food: results Spearman’s r correlation coefficient.

	Liquids	Semi-Solids	Solids
upper right primate	r = 0.28; *p* = 0.183	r = 0.26; *p* = 0.204	r = 0.24; *p* = 0.241
upper left primate	r = 0.02; *p* = 0.929	r = 0.02; *p* = 0.938	r = −0.02; *p* = 0.939
lower right primate space	r = −0.27; *p* = 0.200	r = 0.35; *p* = 0.092	r = 0.07; *p* = 0.731
lower left primate space	r = −0.19; *p* = 0.376	r = 0.24; *p* = 0.149	r = 0.13; *p* = 0.542
maxilar diastema	r = −0.14 *p* = 0.498	r = −0.32; *p* = 0.118	r = 0.25; *p* = 0.237
mandibular diastema	r = −0.03; *p* = 0.870	r = −0.23; *p* = 0.260	r = 0.51; *p* = 0.010 *
terminal plane	r = −0.01; *p* = 0.966	r = −0.24; *p* = 0.243	r = −0.31; *p* = 0.129
overjet	r = −0.64; *p* = 0.001 **	r = −0.49; *p* = 0.014 *	r = −0.08; *p* = 0.717
overbite	r = −0.18; *p* = 0.407	r = −0.23; *p* = 0.289	r = 0.10; *p* = 0.639

* *p* < 0.05 ** *p* < 0.01.

## Data Availability

The data presented in this study are available on request from the corresponding author. The data are not publicly available due to privacy reasons.
